# The Incidence and Characteristics of U.K. Stranger Sex Offenses Fluctuated With Public Health Measures During the COVID-19 Pandemic

**DOI:** 10.1037/vio0000574

**Published:** 2024-12-02

**Authors:** Jessica Woodhams, Blaine Keetch, Prachiben Shah, Matthew Brett, Kari Davies, Heather Flowe, Fazeelat Duran, Sarah Galambos, Pippa Gregory

**Affiliations:** 1 School of Psychology, University of Birmingham; 2 National Crime Agency, London, United Kingdom

**Keywords:** coronavirus, COVID-19, stranger sex offending, rape, routine activity theory

## Abstract

***Objective:*** With COVID-19 came a range of public health measures that impacted people’s routine activities. According to routine activity theory, these could affect the rate and nature of crime. This has largely been examined with volume crime (e.g., burglary, robbery) or crimes committed in the home. Stranger sex offenses greatly vary in nature and occur in a range of settings; therefore, these offenses present a novel opportunity to investigate different routine activity theory-based hypotheses. ***Method:*** The National Crime Agency routinely collects detailed information about all stranger sex offenses reported to the police in the United Kingdom. With these standardized data (*N* = 6,422), we studied the relationship between COVID-19 public health measures and the rate and characteristics of stranger sex offending across the entire first year of the COVID-19 pandemic, contrasting this with data from the same period pre-COVID-19. ***Results:*** Our findings accord with classic criminological theory whereby the incidence and characteristics of U.K. stranger sex offenses reported to police covaried with the population’s patterns of mobility and national lockdowns during the first year of COVID-19. This impact on routine activities also manifested in differences in perpetrator and victim behavior and characteristics. ***Conclusions:*** Our study supports the applicability of routine activity theory to sex offending and brings new insights regarding the situational prevention of sex offending during major events such as a pandemic. It is also relevant to the urgent need to educate prosecutors who are now making decisions about sex offenses perpetrated during the early years of this pandemic.

## Public Significance Statement

Our research shows how changes in individuals’ routine activities because of societal events like a pandemic affects how crimes are committed, against whom, by whom, and when and where they occur. Our study illustrates this with regard to sexual offenses committed by suspects who are strangers to their victims. Our findings are important for theories of sexual offending, how sexual offending is policed, and in planning for future national emergencies.

Established theories from environmental criminology, such as routine activity theory (Cohen & Felson, 1979), would predict that pandemics, such as COVID-19, impact crime rates (Halford et al., 2020) and the nature of offending (Ivandic et al., 2020). Routine activity theory, a classic criminology theory, explains why, when, and where crime occurs: where a motivated offender and suitable target/victim come together in time and space while engaged in routine activities, in the absence of suitable guardians. With the COVID-19 pandemic came a range of public health measures (e.g., stay-at-home orders, closure of hospitality),^1^ and while the pandemic’s impact on other offending, such as domestic abuse, has been studied (Campbell, 2020), its impact on incidence and sex offending patterns has received comparatively little attention owing to limited data, but it should not be overlooked. Preventing sexual violence requires an accurate picture of changing risks, criminal patterns, and behaviors: who is being offended against, how, where, and by whom as pandemics unfold. The purpose of this article, therefore, was to test our hypotheses regarding the impact of the aforementioned public health measures on people’s routine activities, exploring how these changes in routine activities affected where, when, and how sex offenses were subsequently committed and by/against whom during the COVID-19 pandemic, compared to the same period of time pre-COVID-19. The article tests these hypotheses using a large and novel sex offenses data set from the United Kingdom.

In terms of our current understanding of the impact of COVID-19 public health measures on crime, the pandemic’s predicted impacts on crime rates in general have been borne out (Halford et al., 2020; Nivette et al., 2021, although see Mohler et al., 2020, for exceptions). As people’s activities shifted from outside to inside the home, risk of domestic abuse victimization increased while victims’ opportunities to seek and obtain help decreased (e.g., Bradbury-Jones & Isham, 2020). In terms of sex offending rates, a decrease in Mexico City was linked to decreased mobility, particularly in public transport usage, which, prepandemic, was associated with increased risk of sexual victimization (Estévez-Soto, 2021). Scott and Gross (2021) reported a significant decrease in sex offenses in the United States following lockdowns, although not for all types of sexual assaults. The Office for National Statistics (2021) similarly reported in England and Wales an overall decrease in sex offenses from March to May 2020; however, by July to September 2020, rates returned to levels seen in equivalent months in 2019.

A limitation with much of this research, however, including the Office for National Statistics (2021), is that the studies often do not stratify offending by victim–perpetrator relationship. Yet, the nature of the victim–perpetrator relationship can affect location, time, and modus operandi (e.g., Kaufman et al., 1998; Stermac et al., 2004). To our knowledge, to date, only one study has investigated the difference that the COVID-19 pandemic has made on rates of sex offenses, considering victim–perpetrator relationship, and the types of victims and perpetrators involved in these offenses (Spence et al., 2022). This study found that significantly fewer victims aged 13–15 years were targeted, and significantly fewer stranger rape and serious sex offenses were reported, suggesting that the pandemic differentially affected the types of perpetrators and victims involved. However, the data analyzed are from *one* of 43 police forces in the United Kingdom. Given temporal and geographical variations in COVID-19 infection levels and, consequently, differing movement and activity restrictions across the United Kingdom (e.g., differing tiers of restrictions, differences between nations; Jarvis et al., 2021; Laydon et al., 2021), we cannot assume these patterns generalize to the entire United Kingdom.

Alongside affecting crime *rates*, our hypotheses in this article extended to consider how the COVID-19 pandemic and associated public health measures affected the *nature* of sex offenses due to changes in routine activities. Changes in living and working conditions could affect when sex offending occurs as well as behavior exhibited during these offenses. For example, degree of natural surveillance, footfall, and, thus, potential for third-party disturbance can affect modus operandi (Beauregard & Leclerc, 2007). Flowe et al. (2020) investigated sexual violence in Kenya during the COVID-19 pandemic via survivor interviews, noting how it and associated mandates created new situations of sexual violence risk: for children, being home from school, and for adults, having to urinate outside during curfews when fewer witnesses were around. Unfortunately, because data regarding sexual violence are not routinely collected in a standardized manner in Kenya, these qualitative findings could not be confirmed quantitatively.

Stranger sex offenses lend themselves to understanding how changes in routine activities brought about by the COVID-19 pandemic and associated measures might cause changes in crime rates and patterns: By their nature, the two parties (victim and offender) do not cohabit; thus, offenses require the routine activities of both to coincide. Another benefit of focusing on stranger sex offenses is that rich data about crimes these are routinely collated in a standardized manner, on a national scale, by the United Kingdom’s Serious Crime Analysis Section of the National Crime Agency. Rich data about the details of the crimes themselves are rarely available on this scale.

Using stranger sex offenses to investigate how situational changes impact sex offending also addresses a gap in knowledge whereby the relevance of situational factors to promoting risk of sex offending is recognized in psychological theories (Ward & Beech, 2006), but confirmatory empirical research is limited. The COVID-19 pandemic, therefore, presents the opportunity to confirm if situational changes alter offending behavior as theories would predict.

For stranger sex offenses, victims and offenders encounter one another in various settings (Lundrigan et al., 2022), allowing for the investigation of different routine activity theory-based hypotheses. For example, studies report that most stranger sex offenses occur outdoors in a public setting (Lundrigan et al., 2022), yet with national lockdowns leading to greater time inside the home, there should be fewer opportunities for chance encounters outside, leading to a decrease in the number of stranger sex offenses overall during lockdown periods and in specific locations (e.g., on the street). While lockdowns occurred for specified time periods, other public health interventions were less absolute: For example, in the United Kingdom, public houses (pubs)^2^ had periods of complete closure (March 23, 2020, to July 3, 2020, and from November 5, 2020, into 2021), periods of enforced curfew (from September 24, 2020, until the second national lockdown on November 5, 2020), and periods of full reopening (July 4, 2020, to September 23, 2020).

Routine activity theory also has implications for the impact of COVID-19 on the reporting of sex offenses and their commission. For example, in the United Kingdom, the population experienced stay-at-home orders, limits to time allowed outside the home, and requirements to home school children (Jarvis et al., 2021). All of these can inhibit a person’s opportunity to report a sex offense to the police by reducing time outside of the home or by increasing the time needed for caring responsibilities. Qualitative studies of the impact of the COVID-19 pandemic on the policing of sex offenses in the United Kingdom have reported that COVID-19, and lockdowns specifically, led to delayed reporting of offenses to the police. This resulted from victims being unsure about whether travelling to report a sex offense was an “essential reason to travel”^3^ and care demands at home (O’Doherty et al., 2022).

In summary, in accordance with routine activity theory and building on previous studies by exploring in greater detail the behavioral aspects of stranger sex offending, this study assessed how the COVID-19 pandemic and related public health measures were associated with:

1 When stranger sex offending occurred, with the hypothesis that during periods of lockdown, the number of offenses would decrease and that offending rates would be associated with population mobility (Hypothesis 1);
2 Where and when, in space and time, offenders’ and victims’ activities merged for initial encounters to occur;
  a Specifically, we expected lockdowns to change offender/victim mobility, which would decrease the number of initial encounters occurring at some types of location (e.g., on the street, at offender’s or victim’s residence). We also expected the closure of some venues, due to lockdowns and other restrictions on opening, to reduce the number of initial encounters occurring there (e.g., night-time economy venues, such as pubs and nightclubs; Hypothesis 2a);
  b As a consequence of location changes due to COVID-19-related restrictions, we expected the times of stranger sex offending to change particularly that there would be more marked decreases in rates of offending during the weekend (Hypothesis 2b);
3 Offenders’ modus operandi (i.e., what happens during the offense and how is it perpetrated),
  a Specifically, here we expected to see an increase in offenses where the offender wore a mask in the first year of the COVID-19 pandemic compared to the year prepandemic, since mask wearing was encouraged throughout the first year of the pandemic (Hypothesis 3a);
  b Perpetrators use various methods to approach a victim before committing a sex offense, with approach styles available to perpetrators influenced by which situations are conducive to offending, depending on victim availability at different encounter sites (as per routine activity theory). We expected to see a difference in the approach types used by perpetrators based on disruptions to routine activities caused by COVID-19-related restrictions (Hypothesis 3b);
  c As an extension of the changes anticipated in approach type, we also expected to see changes to the use of the internet to facilitate stranger sex offending. While greater internet usage was observed during the pandemic (OpenReach, 2020), therefore offering more opportunities for suspects to first encounter victims online, stay-at-home orders were hypothesized to hinder the prearranged meeting of strangers in person, which would include meetings arranged via the internet (Hypothesis 3c);
4 Delays to reporting, given previously described constraints on movement (and in some cases, increased caring responsibilities), which would increase during lockdown periods (Hypothesis 4);
5 Victim and offender characteristics, using an exploratory, rather than a directional hypothesis approach, because it was unclear how the varied COVID-19-associated public health interventions might affect them.

## Method

### Sample

In the United Kingdom, the Serious Crime Analysis Section of the National Crime Agency is required to receive details of all stranger sex offenses reported to the police, including rape, attempted rape, and indecent or sexual assault offenses meeting their analysis criteria (see Section 2.2, College of Policing, 2012). Information about these offenses are input into two systems: (a) the Serious Crime Analysis Section Information Database (SID), which records basic case information on all submissions made to the unit, and (b) the United Kingdom’s version of the Violent Crime Linkage Analysis System (ViCLAS; Collins et al., 1998), a database used worldwide to assist in behavioral crime linkage of stranger sex offenses and which contains a detailed breakdown of offense behavior and contexts across many variables. Both data sets are used in this study to provide different information: SID is a larger data set, while ViCLAS has much more case detail. For a variety of reasons, not every case recorded on SID makes it into ViCLAS. Excluded cases include those where there is a lack of victim engagement or poor recollection, meaning sufficient detail is not available to index the case into ViCLAS; in other words, all ViCLAS offenses in this study were also in the SID data set, but not all the SID data were in the ViCLAS data set. In total, we received 4,905 cases from SID (3,030 pre-COVID-19, 1,875 peri-COVID-19) and 1,517 cases from ViCLAS (981 pre-COVID-19, 536 peri-COVID-19). The ViCLAS cases involved 2,109 offenders (1,378 pre-COVID-19, 726 peri-COVID-19) and 1,559 victims (1,012 pre-COVID-19, 547 peri-COVID-19).

### Procedure

The study received ethical approval from the University of Birmingham science, technology, engineering, and mathematics Ethics Committee (ERN_11-0098B) and was also approved by the National Crime Agency’s Research Approval Panel. The Serious Crime Analysis Section provided anonymized data, securely transferred using Criminal Justice Secure Mail accounts. Geographical information was omitted, preserving the anonymity of all parties involved. The data used in this article were provided to the authors on the basis that it would be kept confidential; therefore, the data cannot be shared more widely.

### Measures

For each offense, several details were provided: the date of offense, date reported, type of initial encounter location, victim and perpetrator demographic characteristics (e.g., age, gender, ethnicity) where available, and detailed information about perpetrator behavior (e.g., if the offense was internet-facilitated, i.e., the internet was used to prearrange a meeting in person, or if the offender wore a mask). For both databases, data were provided in binary format, where 1 denominates the presence of any one variable and 0 its absence or were continuous (i.e., offender and victim age). Delays in reporting were calculated in days by calculating the number of days between the date of offense and date reported. The SID data were selected for the analyses where the required variables were available because this was the larger data set; where variables only existed in ViCLAS, ViCLAS data were used.

Additionally, for Hypothesis 1, a measure of population mobility was required. This was created from Google data obtained online (https://www.google.com/covid19/mobility). Within these data are separate measures of percentage change in daily visits for categories of activity—retail and recreation, grocery and pharmacy, parks, transit stations, workplaces—compared to Google’s baseline day. A percentage change value is only available from January 14, 2020. For “residential,” it is the time spent at a residential location per day compared to the baseline day. “A baseline day represents a *normal* value for that day of the week. The baseline day is the median value from the 5-week period Jan 3 – Feb 6, 2020.” From the daily values, we calculated a mean monthly value.

### Data Analysis Plan

Before analysis, data were preprocessed to mitigate potential errors in the inputting of data onto the data set using Pandas, a specialized, open-source software library for data manipulation and analysis that is built on top of the Python programming language (https://pandas.pydata.org/). Such errors included duplicated entries and erroneous entries, such as when the date an offense was reported to the police was recorded as a date that fell before the offense date or where both options for a two-category response were selected as positive (e.g., for a two-category gender variable, where both male and female were selected). Cases with such errors were removed from our sample and do not feature in our results.

To compare the overall offending prevalence to population mobility as part of Hypothesis 1, the data were graphed using Pandas with no formal hypothesis testing. This method was chosen as insufficient pre-COVID-19 data exist for the Google mobility data to enable inferential statistical analysis.

To assess differences in sex offending between lockdown and nonlockdown periods, as outlined in Hypotheses 1, 2, and 3, date of offense was used to create designated time periods. The lockdown periods were designated based on official lockdown months (i.e., March–May 2020 inclusive, November–December 2020 inclusive, and January–February 2021 inclusive) since daily data contained many short-term fluctuations that were not relevant to the overall trends and patterns (Box & Tiao, 1975; McDowall et al., 2019). For Hypothesis 2 and our location testing, a different period of intervention was defined for the night-time economy since nightclubs did not reopen in the United Kingdom during our data period, and pubs were subject to additional restrictions. We therefore defined two “lockdown” periods for the night-time economy—the first follows the first U.K. national lockdown months (March–May 2020 inclusive) and the second dates from September 2020 to February 2021 encapsulating the curfews imposed on pubs in September/October 2020 and the remaining two national lockdowns, since pubs did not reopen in the gap between national Lockdowns 2 and 3.

Autoregressive integrated moving average (ARIMA) analyses were employed to examine how lockdown measures affected outcomes over time. Although no standardized form of power analysis has been established for ARIMA analyses (Hawley et al., 2019), greater power is associated with more data points, more observations per data point, and an absence of autocorrelation (Dorais, 2024). A minimum of nine data points pre- and postintervention and, where relevant, *between* interventions is recommended (Jandoc et al., 2015). As this is a naturalistic ARIMA analysis, we could not dictate when the interventions occurred; therefore, we have exceeded the recommendation for minimum preintervention data points (with 12 data points), but there were multiple lockdowns during the first year of the COVID-19 pandemic (three lockdowns) and none had nine time points (months) between them, despite us having 12 data points (months) in total peri-COVID-19. Other studies of the impact of COVID-19 on crime do not have 12 data points postintervention (e.g., Langfield et al., 2021; Payne et al., 2022). Regarding power and number of observations, a minimum of 50 observations is recommended (Dorais, 2024), and other studies on the impact of COVID-19 on crime have used 197 observations (Kim & Phillips, 2021). Our ARIMA analyses have between 210 and 4,905 observations. Regarding observations per data point, our numbers mirror other ARIMA analyses of sex offenses (e.g., Lightowlers et al., 2023, had four to 23 observations per data point whereas we had one to 275).

ARIMA analyses were conducted using IBM’s SPSS for Statistics 27, where dummy variables were created to differentiate between pre- and peri-lockdown periods for each outcome variable. The outcome variables were: (a) number of offenses; (b) number of offenses occurring on a weekend; (c) number of offenses where the victim and offender encountered each other in a night-time economy venue, (d) at the offender residence, (e) at the victim residence, or (f) on the street; (g) number of offenses that were internet facilitated; and (h) number of offenses where the offender sneaked up on the victim, (i) engaged the victim in conversation, or (j) arranged to meet the victim. Descriptive statistics were calculated for each outcome variable for pre-and peri-lockdown (Table 1). ARIMA specifies its models by three parameters: where “*p*” (autoregression model) captures autocorrelation by incorporating lagged values of the dependent variable, “*d*” represents the number of differencing steps needed to achieve stationarity in the time series data, and “*q*” (moving average model) captures moving average to account for noise and irregularities in the data. SPSS Expert Modeler was used to determine the most suitable ARIMA (*p*, *d*, *q*) model for each outcome variable. The notation of 1 or 0 in parentheses, for example, (0, 1, 1) indicates which of the parameters were used in specifying the model (with a 1 indicating a parameter’s use). The Ljung–Box test for all the variables showed that the residuals had no remaining autocorrelation (Dorais, 2024; Li et al., 2023).[Table tbl1]

For analyses of reporting delays as part of Hypothesis 4, we used the actual national lockdown dates to designate the lockdown periods (Lockdown 1: March 23, 2020, to May 13, 2020; Lockdown 2: November 5, 2020 to December 1, 2020; Lockdown 3: January 5, 2021 to February 28, 2021).

Where pre-peri COVID-19 comparisons were made in Hypothesis 3a and 4, pre-COVID-19 was from March 1, 2019, to February 28, 2020, and peri-COVID-19 was from March 1, 2020, to February 28, 2021. March 1, 2020, was chosen because the U.K. government implemented social distancing measures before national lockdowns to tackle COVID-19’s spread (e.g., advice to avoid pubs was given on March 16, 2020). We used 2 × 2 chi-square analyses to assess the association between pre- versus peri-COVID-19 and (a) if the offense was internet-facilitated or not and (b) if the offender wore a mask or not. Skewed distributions (Field, 2009) meant that Mann–Whitney *U* tests and Kruskal–Wallis tests, computed using IBM’s SPSS Statistics 27, were used to assess differences in reporting delays between lockdown periods and no-lockdown periods (Hypothesis 4; Supplemental Material).

Finally, exploratory analyses of differences in victim and offender characteristics across the pre- versus peri-period were computed using Pandas.

## Results

### Hypothesis 1: The Prevalence of Stranger Sex Offending

#### Rates of Stranger Sex Offending (SID Data)

**Figure 1 fig1:**
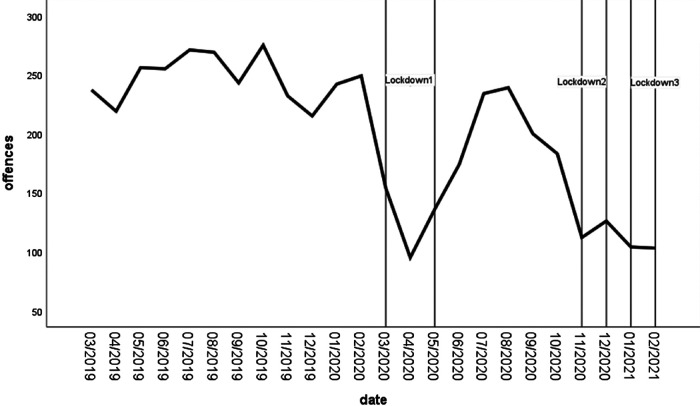
Number of Stranger Sex Offenses Occurring per Month 1 Year pre-COVID-19 and During the First Year of COVID-19 *Note*. Lockdown periods are displayed as vertical bars.

**Figure 2 fig2:**
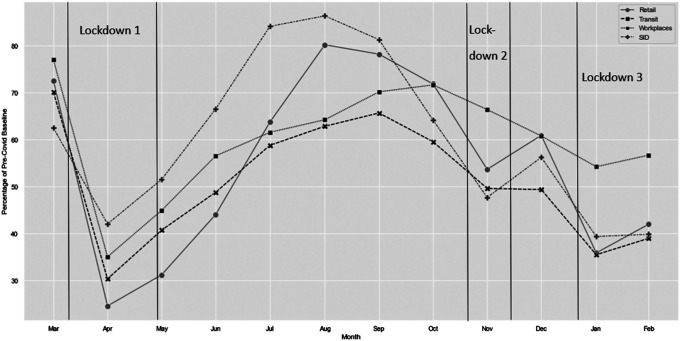
Stranger Sex Offenses in COVID-19’s First Year (March 2019–February 2020) as a Proportion of Those Occurring Pre-COVID-19 and Monthly Change in Google’s Mobility Data *Note*. Stranger sex offenses in COVID-19’s first year (March 2019–February 2020) as a proportion of stranger sex offenses occurring during the same months pre-COVID-19 (dashed line with plus) and the monthly change in Google’s mobility data for visits to places associated with retail and recreation (solid line with circles), transit stations (dashed line with crosses), and workplaces (dotted line with squares) are displayed as a proportion of the baseline day. Lockdown periods are displayed as vertical bars. These findings are descriptive, as opposed to statistically tested. SID = Serious Crime Analysis Section Information Database.

The ARIMA model (0, 1, 0) accounted for 42% of the variability attributed to Lockdowns 1, 2, and 3 (see Table 1; the results of all the ARIMA analyses are presented here). As per Hypothesis 1, Lockdown 1 was significantly associated with a 66.50 reduction in offenses (*t* = −3.05, *p* < .001), Lockdown 2 with a reduction of 71.00 (*t* = −2.30, *p* < .05), and Lockdown 3 with a 93.09 reduction (*t* = −2.13, *p* < .05; Figure 1). Using Google mobility data, we investigated whether the rate of stranger sex offending (as reported to the police and recorded in SID) was associated with population mobility during the first year of the COVID-19 pandemic. As per routine activity theory, the rate of offending tracked population mobility (Figure 2). Decreases in offending tracked decreases in population mobility specifically related to retail and recreation activities. These findings are descriptive rather than statistically significant.[Fig fig1][Fig fig2]

### Hypothesis 2: Where and When Stranger Sex Offending Occurs

#### Hypothesis 2a: Where Stranger Sex Offending Occurs (ViCLAS Data)

**Figure 3 fig3:**
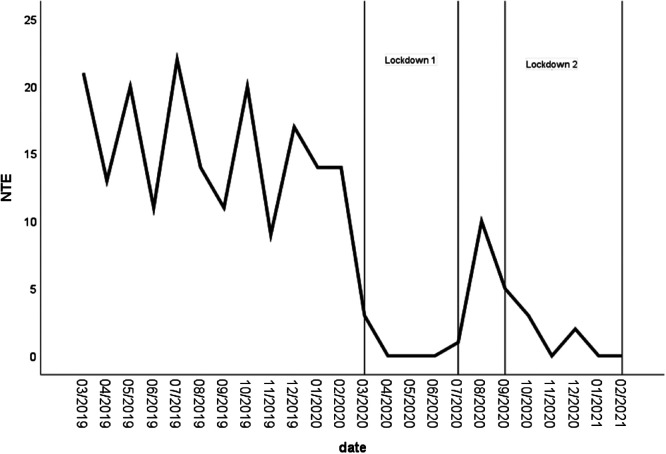
Number of Stranger Sex Offenses Pre-COVID-19 and During the First Year of COVID-19 Where the Encounter Site Was a Night-Time Economy (NTE) Venue *Note*. Lockdown periods are displayed as vertical bars.

In line with Hypothesis 2a, the ARIMA analysis (1, 1, 0) for offenses where the victim and offender encountered one another in a night-time economy venue accounted for 67% of the variability attributed to the two lockdown periods (Figure 3). Lockdown 1 was significantly associated with an 11.51 reduction (*t* = −5.47, *p* < .001) and Lockdown 2 with a 7.20 reduction (*t* = −2.42, *p* < .05).[Fig fig3]

For initial encounter sites in the offender’s residence, the ARIMA analysis (1, 1, 0) accounted for 58% of the variability attributed to the three lockdowns, but only the reduction for the first lockdown (a reduction of 9.58) was significant (*t* = −2.45, *p* < .05). The reductions for Lockdowns 2 and 3 were smaller (3.50, *t* = −0.58, *p* > .05; 2.54, *t* = −0.22, *p* > .05, respectively). In contrast, for initial encounter sites in the victim’s residence, only the reduction of 7.26 for Lockdown 3 was significant (*t* = −3.27, *p* < .001). The ARIMA model (0, 0, 0) accounted for 39% of the variability attributed to the lockdowns. The reductions for Lockdowns 1 and 2 were 2.43 (*t* = −1.30, *p* > .05) and 3.76 (*t* = −1.69, *p* > .05).

**Figure 4 fig4:**
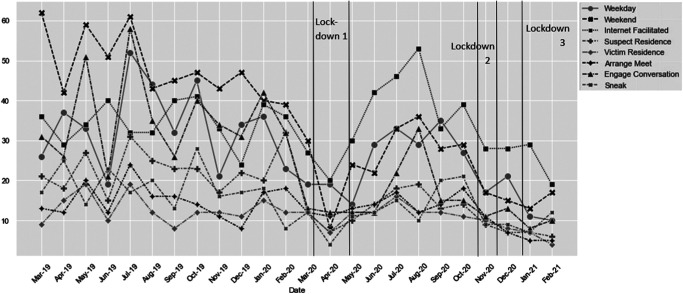
Number of Stranger Sex Offenses Occurring per Month 1 Year pre-COVID-19 and During the First Year of COVID-19 (for Multiple Variables) *Note*. Lockdown periods are displayed as vertical bars.

The ARIMA analysis (0, 0, 0) for initial encounters on the street accounted for 57% of the variability attributed to the three lockdowns. As expected, according to routine activity theory, all three lockdowns denoted significant reductions in offenses (Lockdown 1: 10.31, *t* = −2.85, *p* < .05; Lockdown 2: 13.64, *t* = −3.15, *p* < .05; Lockdown 3: 16.64, *t* = −3.85, *p* < .001). See Figure 4 for a visual representation of the location of offenses occurring each month.[Fig fig4]

#### Hypothesis 2b: When Stranger Sex Offending Occurs (ViCLAS Data)

The changes to when offenses occurred were explored using two ARIMA analyses. The ARIMA (1, 0, 0) model (Figure 4) for offenses occurring on the weekend accounted for 57% of the variability attributed to Lockdowns 1, 2, and 3. Lockdown 1 was significantly associated with a 22.09 reduction in weekend offenses, Lockdown 2 with a 26.76 reduction, and Lockdown 3 with a 27.76 reduction (*t* = −22.09, *t* = −26.76, *t* = −27.76, respectively, all significant at *p* < .001). The ARIMA model (1, 1, 0) for weekday offending accounted for 38% of the variability attributed to the three lockdowns. However, the reductions were not significant (−1.41, −6.55, and −6.52).

### Hypothesis 3: The Modus Operandi of Stranger Sex Offending

#### Hypothesis 3a: Mask Wearing (ViCLAS Data)

As the pandemic continued in the United Kingdom, wearing a face mask or face covering became mandatory in public places (e.g., Blackburn, 2020). The subsequent increased wearing of face masks by the public in COVID-19’s first year is borne out in our data. Mask wearing was not limited to lockdown periods in the first year of COVID-19; therefore, a 2 × 2 chi-square analysis was conducted with mask wearing by the offender (yes, no) and pre-COVID-19/peri-COVID-19 as the other variable. A significantly greater proportion of offenders wore masks to offend during the first year of COVID-19 (4.13%, *n* = 30) compared to pre-COVID-19 (1.16%, *n* = 16); however, the effect size was small (*N* = 2,109, χ_2_ = 18.38, *p* < .0001, Φ = .09).

#### Hypothesis 3b: Initial Approach (ViCLAS Data)

To assess changes to approach types, we conducted three ARIMA analyses, one for each of three types of approach measured in this study—offender arranging to meet the victim, offender engaging the victim in conversation, and offender sneaking up on the victim (Figure 4). Arranging to meet the victim was impacted little by the lockdowns as indicated by the ARIMA analysis (0, 1, 0), only accounting for 12% of the variability attributed to lockdowns and with no-lockdown period, resulting in a significant reduction in offenses (with reductions of 3.50, 7.04, and 9.09, respectively). The ARIMA model (0, 1, 0) for engaging the victim in conversation accounted for 43% of the variability attributed to lockdowns. Reductions in offenses for all three lockdowns were significant and were 18.49 (*t* = −2.65, *p* < .05), 18.82 (*t* = −2.26, *p* < .05), and 21.82 (*t* = −2.62, *p* < .05), respectively. The ARIMA model (0, 1, 0) for sneaking up on the victim accounted for 40% variability attributed to lockdowns. Again, reductions in offenses for all three lockdowns were significant and were 8.29 (*t* = −2.67, *p* < .05), 8.29 (*t* = −2.24, *p* < .05), and 7.79 (*t* = −2.10, *p* < .05), respectively.

#### Hypothesis 3c: Internet-Facilitated Offenses (SID Data)

We tested the effect of lockdowns on the number of internet-facilitated offenses using an ARIMA analysis. Lockdown 1 was significantly associated with a 11.33 reduction in offenses (*t* = −2.80, *p* < .05); Lockdown 2, a shorter lockdown, was associated with a nonsignificant 9.00 reduction in offenses; and Lockdown 3 was associated with a significant 13.00 reduction in offenses (*t* = −2.70, *p* < .05). The ARIMA model (0, 0, 0) accounted for 43% of the variability attributed to the three lockdowns. See Figure 4 for these data plotted by month.

### Hypothesis 4: Delays in Reporting (SID Data)

**Figure 5 fig5:**
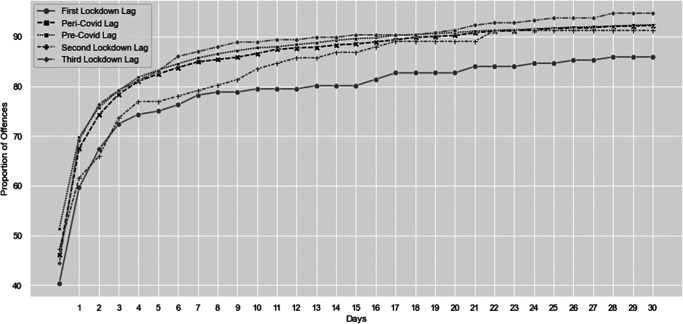
Proportion of Stranger Sex Offenses Reported to Police From 0 Days to 30 Days Pre-COVID-19, Peri-COVID-19, and for Each of the Three Lockdowns

As hypothesized, our data demonstrated that, compared to pre-COVID-19, stranger sex offenses were less likely to be reported on the same day as the offense during all three lockdown periods. However, this pattern was far less pronounced for the second and third national lockdowns (Figure 5). A Mann–Whitney *U* test comparing the number of days between the offense date and the reporting date (the reporting delay) for pre- (*n* = 2,949) and peri-COVID-19 (*n* = 1,866) showed a significant increase with COVID-19 (*U* = 2904458.00, *z* = 3.49, *p* < .001, *d*^4^ = .09). The median reporting delay peri-COVID-19 was 1.00 compared to a median of 0.00 pre-COVID.[Fig fig5]

We compared reporting delays across four periods during the first year of COVID-19: the three lockdown periods and the no-lockdown period. A Kruskal–Wallis test revealed significant differences in reporting delays across these periods, *H*(3, *n* = 4,815) = 11.87, *p* < .01, *d* = .09 (Footnote 4). Pairwise comparisons, however, showed that reporting delays were significantly longer during the first lockdown compared to the no-lockdown period. Differences between the other lockdowns and the no-lockdown period were not significant. The median reporting delay for each of the four time periods (including no lockdown) was the same (1.00 day). The mean reporting delay during the lockdown periods decreased across the year (21.58 days for Lockdown 1, 16.05 for Lockdown 2, and 14.70 for Lockdown 3), compared to a delay of 11.80 days during the no-lockdown period; however, it is important to note that the data were skewed.

### Exploratory Research Question: Victims and Offenders of Stranger Sex Offending (ViCLAS Data)

In the pre-COVID-19 sample, there were 1,012 victims compared to 547 victims in the peri-COVID-19 sample. Victim gender was known for 1,010 and 545 victims, and most victims in both time periods were female (93.37%, *n* = 943 and 91.01%, *n* = 496, respectively). For all other victims where gender was recorded, they were male. During both time periods, most victims were of White ethnicity (pre: 83.37%, *n* = 762; peri: 82.51%, *n* = 401). Most offenses involved a lone victim (pre: 96.64%, peri 96.08%).

**Figure 6 fig6:**
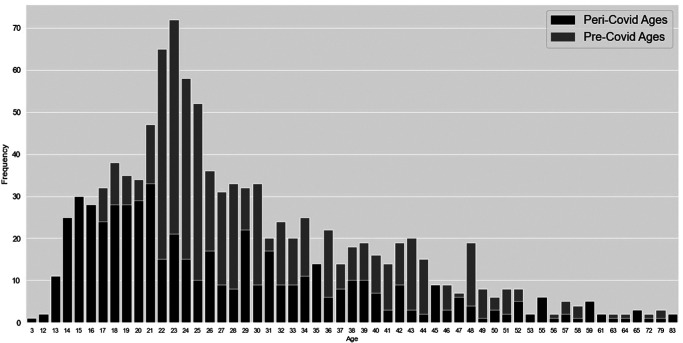
The Distribution of Victim Ages in Years for 1 Year Pre-COVID-19 and 1 Year Peri-COVID-19 *Note*. This figure represents descriptive, as opposed to statistically tested, trends in the data.

Given the large impact of the pandemic on the night-time economy, meaning fewer victims would be accessible in such settings, and the age profile associated with the night-time economy (Bourdeau et al., 2017), it was not surprising to see a decrease in offenses against victims in their late teens and early 20s peri-COVID-19 (Figure 6; this decrease is descriptive as opposed to statistically significant). The distribution of victim ages peri-COVID-19 is similar to pre-COVID-19; however, a sizeable proportion of victims in their early 20s are missing from the peri-COVID-19 distribution.[Fig fig6]

**Figure 7 fig7:**
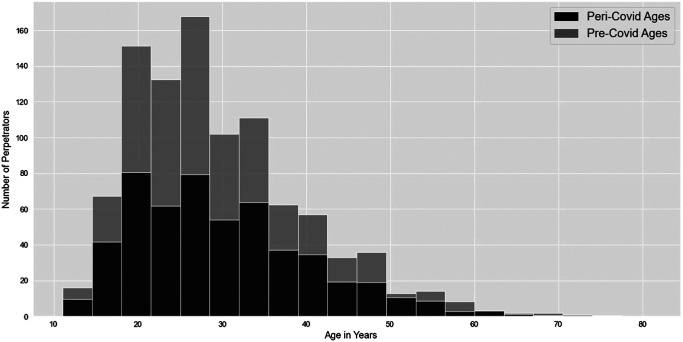
The Distribution of Perpetrator Ages in Years for 1 Year Pre-COVID-19 and 1 Year Peri-COVID-19 *Note*. This figure represents descriptive, as opposed to statistically tested, trends in the data.

In the pre-COVID-19 sample, there were 1,378 offenders and 724 in the peri-COVID-19 sample. Most offenses involved a lone offender (pre: 77.17%, peri: 77.80%). In both samples, most offenders were male (pre: 99.41%, *n* = 1,349; peri: 98.88%, *n* = 709). All others were recorded as female where gender was known. In both time periods, most offenders were of White European ethnicity (pre: 47.51%, *n* = 458 of 964; peri: 51.47%, *n* = 262 of 509). Regarding age, like victims, a large proportion of offenders in their mid- to late 20s were missing from the peri-COVID-19 distribution (Figure 7; again, this decrease is descriptive and has not been statistically tested), which likely reflects the reduction in offending associated with the night-time economy and the age groups associated with such venues. As per routine activity theory, with their closure, there are reduced opportunities for offenders and victims in those age ranges to come together in space and time.[Fig fig7]

## Discussion

Using a large, standardized stranger sex offenses data set spanning a full year before COVID-19 and its first year, we investigated the pandemic’s impact on sex offense characteristics, including timing behaviors, and locations in the United Kingdom. Significantly fewer stranger sex offenses occurred during lockdown periods as hypothesized. During lockdown periods, there was reduced offending in night-time economy and street locations, with some reduction in home locations. Weekend offending also significantly decreased. With regard to the modus operandi hypotheses, a small but significant increase in offender mask wearing was seen. Offenses involving offenders engaging victims in conversation or sneaking up on them decreased significantly during lockdowns, as did internet-facilitated offenses in two of the three lockdown periods. Last, our exploration of victim and offender characteristics revealed a decrease, descriptively speaking, in the number of victims and offenders in their late teens and early 20s in offenses committed during, as opposed to pre-, the COVID-19 pandemic.

Our data supported the hypothesis that stranger sex offending rates fall during periods of lockdown due to reduced opportunities for offender(s)–victim interactions as per routine activity theory. What is difficult to disentangle in our data is whether the pandemic led to less offending per se or a reduction in victims reporting to the police or both. Qualitative reports from the United Kingdom and Kenya (Flowe et al., 2020; O’Doherty et al., 2022) suggest that some victims chose not to report to the police where they had been assaulted while breaching lockdown restrictions (e.g., curfews). However, findings from a national qualitative study of practitioners from the U.K. Sexual Assault Referral Centres, which provide support to victims without police involvement and which, therefore, do not carry risk of punishment for breaching lockdown rules, suggest that a reduction in victim reporting to police may not be the full explanation (Justice in COVID-19 for Sexual Abuse and Violence, 2022). This study reported a reduction in offenses coming to their attention that occurred in night-time economy settings and on public transport during lockdown, suggesting that offenses occurring at these locations decreased.

Since the weekends afford most people with greater unstructured time, we expected that the lockdowns might have a greater effect on the number of offenses occurring on weekends compared to weekdays. We tested this and found this to be the case. By graphing the rate of stranger sex offending alongside Google mobility data, we could see that the rate of offending tracked population mobility for retail and recreation activities, in particular.

Night-time economy-associated offending decreased with venue closure, lockdowns, and enforced curfews as did offenses that began with an initial encounter on the street, as expected. Lockdown-associated reductions in offenses were also observed for offenses where the victim and offender encountered each other at one another’s residence. For such offenses, the victim and offender can have initially engaged with one another via media (e.g., internet, phone), but their first physical encounter for the offense is at a residence. We would expect to see a reduction with lockdowns since the directive with a national lockdown was that people should not be mixing in the home with people with whom they do not normally live. Research has documented a decline in adherence to mixing rules across the three lockdowns (Ross et al., 2021), and this could explain why only Lockdown 1 resulted in a significant reduction in offenses associated with an encounter at the offender’s residence; however, it would not explain the pattern seen for victim residence where the opposite pattern was observed with only the reduction for Lockdown 3 being significant.

During the pandemic, the U.K. population spent more time on the internet (OpenReach, 2020), again, reflecting a shift in routine activities for victims and perpetrators, providing greater opportunity to encounter one another online. When comparing the proportion of offenses that were internet facilitated pre-COVID-19 to peri-COVID-19, we found a significant association with more reported during the first year of COVID-19. However, offenses facilitated by the internet were still impacted by two lockdowns since, following this initial meeting online, the victims and offender must come together physically in space and time for the offense to occur, and lockdowns would have made this more difficult.

Our exploratory analysis of whether routine activity changes might alter who was at risk of sexual victimization during the COVID-19 pandemic identified an apparent reduction in the number of victims in their late teens/early 20s during COVID-19’s first year. We attribute this observation, at least partly, to night-time economy venues closing and, thus, a reduction in offenses associated with this age group since, pre-COVID-19, victims of these ages are associated with night-time economy-related offenses. Similarly, we observed an apparent reduction in offenders aged in their mid–late 20s.

In addition to gaining insights into COVID-19’s impact on offender behavior, we examined how victims’ reporting behavior changed. Specifically, we examined whether COVID-19 led to greater delays in reporting to the police. Fewer offenses were reported on the same day as the offense during COVID-19’s first year compared to the previous year. This was particularly pronounced in the first lockdown where people’s movements were more restricted than in subsequent lockdowns and when there had been little time to adapt to additional caring responsibilities, potentially inhibiting victim reporting. The sudden contraction of social networks, which support victims’ reporting (Paul et al., 2014) or victims’ concerns about reporting had they been in breach of lockdown rules, could also lead to delays. Our analyses confirmed that increases in delays to reporting were significant pre- compared to peri-COVID-19, and also when comparing the first lockdown to time periods of no lockdown in the same year.

### Limitations

Our data represent offenses reported to the police with known underreporting rates. Only 16% of rapes are reported to police, with younger (Office for National Statistics, 2021), male (Javaid, 2018), homosexual (Mitchell et al., 1999), and non-White victims (Bergenfeld et al., 2022) additionally underrepresented. Further, victim accounts of offenses to the police may contain errors and omissions due to embarrassment, shame, or difficulties recalling aspects of an offense (Alison et al., 2001).

### Future Research Directions

Victimization survey data would help determine whether the ratio of offenses occurring, compared to offenses reported to the police, changed during lockdowns. Further, while stranger sex offenses are more likely to be reported to police (Paul et al., 2014), there will still be underreporting. ViCLAS collates more information about the offense than victimization surveys; therefore, new victimization surveys would need to be designed that include questions to gather detailed information about offender behavior while ensuring victims have appropriate support during and after the survey. Alternatively, Sexual Assault Referral Centres could conduct surveys in this regard with victims who have given informed consent to participate in a research study. Replication studies in other countries using equivalent ViCLAS data to our U.K. data would also be valuable.

### Prevention and Policy Implications

Our findings indicate to whom and in what circumstances stranger sex offenses occurred pre- and during a pandemic. This knowledge is relevant to policing these offenses and to policies aimed at preventing sexual violence. For example, knowing that sex offending appears to be linked to the routine activities of victims and offenders (i.e., a situational influence) suggests that we should consider how situational interventions could be effective in addition to intervening with perpetrators once apprehended. It allows the police to justify resources being put into policing events or geographical areas where activities bring a large volume of potential victims and offenders together (e.g., night-time economy areas, festivals, and other major cultural events).

Policymakers can also use our findings, alongside those of other authors, in planning for future major national events like a pandemic by considering what resources are needed and how services would need to respond. Delayed reporting has been a reason for negative assessments of victim credibility (e.g., Jordan, 2004); therefore, educational efforts to challenge such perceptions are needed if the national emergency itself, or the interventions put in place, makes it harder for victims to report quickly. Our findings highlight the need to financially resource and ensure the operational readiness of remote options for reporting sex offenses (and other crimes), as well as thinking carefully about the messaging to victims wanting to report who may be deterred because of lockdown rules. In our data, on average, lockdowns resulted in the delay of a day in reporting the offense to the police. Forensic evidence of a sexual assault deteriorates quickly (Kane et al., 2021); therefore, other means to promptly gather forensic evidence that do not require travel must be developed.

Last, our findings provide an important context to police officers responding to delayed reports of sex offenses experienced by victims during the pandemic and to prosecutors who are presently making decisions about the progression of sex offenses through the criminal justice process that were committed during the first year of the COVID-19 pandemic. As a result, our findings have been shared already with the National Police Chief’s Council and the Crown Prosecution Service in the United Kingdom.

### Conclusion

Changes in social behavior and mobility in the United Kingdom are reflected at a broad level in changes in the rate, locations, and offender behavior in stranger sex offending. Our study provides evidence for routine activity theory’s relevance to sex offending and illustrates when and where victims are vulnerable to sex offending during and outside of a pandemic, bringing new insights to the situational crime prevention of sex offending. Our findings also provide an indication of how sex offending may play out in relation to public health measures that are adopted in response to future crises, be they natural disasters, pandemics, or large-scale conflicts.

## Supplementary Material

10.1037/vio0000574.supp

## Figures and Tables

**Table 1 tbl1:** Descriptive Statistics for the Outcome Variables Before and During Lockdown Period(s)

Outcome variable	Lockdown 1			Lockdown 2			Lockdown 3		
	*M*_pre_	*M*_peri_	Point change	*M*_pre_	*M*_peri_	Point change	*M*_pre_	*M*_peri_	Point change
Sexual offenses (*n* = 4,905)	211.33	128.33	−83.00	208.41	119.00	−89.41	209.82	103.50	−106.32
Night-time economy encounter site (*n* = 210)	10.85	0.80	−10.05	11.11	1.66	−9.45			
Offender residence encounter (*n* = 410)	18.14	9.66	−8.48	17.90	8.00	−9.90	12.06	5.50	−6.56
Victim residence encounter (*n* = 277)	11.71	10.33	−1.38	11.77	9.00	−2.77	18.00	4.00	−14.00
Street encounter (*n* = 404)	17.76	10.33	−7.43	17.72	7.00	−10.72	14.09	5.00	−9.09
Offender arranged to meet victim (*n* = 320)	13.52	12.00	−1.52	13.72	9.00	−4.72	26.59	9.00	−17.59
Offender engaged victim in conversation (*n* = 603)	26.95	12.33	−14.62	26.31	12.00	−14.31	15.40	9.50	−5.90
Offender sneaked up on victim (*n* = 358)	15.76	9.00	−6.76	15.45	9.00	−6.45	34.63	24.00	−10.63
Internet-facilitated offenses (*n* = 810)	34.90	25.66	−9.24	34.27	28.00	−6.27	29.31	10.50	−18.81
Weekday offenses (*n* = 210)	29.23	17.33	−11.90	28.54	19.00	−9.54	37.31	15.00	−22.31
Weekend offenses (*n* = 666)	37.57	20.66	−16.91	37.22	16.00	−21.22	8.18	2.00	−6.18
*Note*. Mean refers to the mean number of offenses, and point change refers to the increase or decrease in number of offenses. The *n* indicates the number of offenses overall that were applicable for the category of outcome offense (e.g., 404 offenses occurred on the street which represents the sample for this analysis).									

## Footnotes

1 A visual display of the public health interventions implemented in the United Kingdom and their timescales can be seen at https://www.instituteforgovernment.org.uk/data-visualisation/timeline-coronavirus-lockdowns.

2 Qualitative research has documented a clear association between night-time economy venues and sexual assaults in the United Kingdom, particularly when alcohol is consumed (e.g., Christmas & Seymour, 2014).

3 During lockdowns, the U.K. Government required citizens to engage in only “essential” travel (Sky News, November 2, 2020, https://news.sky.com/story/coronavirus-travel-rules-after-england-goes-into-second-lockdown-on-thursday-12120410).

4 *d* was calculated here using https://www.psychometrica.de/effect_size.html.
