# Supplementary material for: The Incidence and Characteristics of U.K. Stranger Sex Offenses Fluctuated With Public Health Measures During the COVID-19 Pandemic
Source: Psychol Violence. 2024 Dec 2;15(4):502–14. doi: 10.1037/vio0000574 (PMC12203417; doi:10.1037/vio0000574)
Supplement: Supplementary file 1 [file VIO-2023-1092_supplemental_materials.docx]

**Sensitivity Analyses for ‘The Incidence and Characteristics of UK Stranger Sex Offenses Fluctuated with Public Health Measures During the Covid-19 Pandemic’**

Sensitivity analyses or power analyses are not yet available for ARIMA analyses. Therefore, we report here on sensitivity analyses for the 2x2 chi-square tests, Mann-Whitney U test and Kruskal-Wallis test reported in our manuscript.

**Mann-Whitney U test for Delays in Reporting**

A sensitivity analysis computed using G*Power 3.1.9.7 indicated that with an alpha value of 0.05, power of 0.95 and sample sizes in each group of 2,949 and 1,866 for pre-Covid-19 and peri-Covid-19, respectively, the required effect size would have been .11.

**Kruskal-Wallis test for Delays in Reporting**

A sensitivity analysis computed using G*Power 3.1.9.7 for a one-way ANOVA (calculated because there is not an option for Kruskal-Wallis in this program) indicated that with an alpha value of 0.05, power of 0.95 and an overall sample size of 4,815 offenses, the required effect size would have been .06.

**Chi-square analysis for Internet-facilitated Sexual Offending**

A sensitivity analysis computed using G*Power 3.1.9.7 for a chi-square test with an alpha value of 0.05, power of 0.95, an overall sample size of 4,905 offenses and 1 degree of freedom, indicated that the required effect size would have been .05.

**Chi-square analysis for Mask-wearing**

A sensitivity analysis computed using G*Power 3.1.9.7 for a chi-square test with an alpha value of 0.05, power of 0.95, an overall sample size of 2,109 offenders and 1 degree of freedom, indicated that the required effect size would have been .08.
